# Editorial: Shaping with data: Using pharmacoepidemiology to shape pharmaceutical policy and clinical decision-making

**DOI:** 10.3389/fphar.2022.1023304

**Published:** 2022-10-12

**Authors:** Mina Tadrous, Andrea Burden

**Affiliations:** ^1^ Leslie Dan Faculty of Pharmacy, University of Toronto, Toronto, ON, Canada; ^2^ Department of Chemistry and Applied Biosciences, Institute of Pharmaceutical Sciences, ETH Zurich, Zurich, Switzerland

**Keywords:** big data, pharmacoepidaemiology, drug policy, pharmaceutical services, observational studies

Policymakers around the world are grappling with an influx of rapidly changing science and new treatments in the area of medications and health technology. Much of their decisions have historically relied on randomized controlled trial (RCT) data for drug assessments. Over the past decade, it has become important for many decision-makers to realize that the RCT design may not reflect real-life clinical practice as the trial populations may exclude important patients seen in clinical practice. More importantly, the RCT is unable to answer questions related to rare adverse events, optimal use, and access. Therefore, the use of real-world data in the field of pharmacoepidemiology has, in many cases, stepped up to help policymakers fill these gaps. In this special topic, we aimed to gather a global cross-section of various papers that showcase the power of pharmacoepidemiology in helping shape policy and clinical practice.

We set out to cover the many areas that pharmacoepidemiology can be used to shape and inform policymakers, including understanding beneficial and adverse drug effects of medications, drug utilization, real-world effectiveness, clinical effects of drug-drug interactions, effects of medication non-adherence, and the impact of policy changes on drug utilization. We were able to do just that and covered many of these topic areas. Excitingly we received work from over eight different countries, each leveraging unique data sources and study designs that truly highlight the breadth of work that can happen in this field. Most importantly we received work that showcased the ability for pharmacoepidemiology to be used in studying drug safety, policy, and clinically relevant questions.

Specifically, we included a number of exciting papers on the safety of drugs with important clinical applications in the area of anticoagulants (Perreault et al.), drug-drug interactions between methadone and antidepressants (Antoniou et al.), use of antiseizure medications among pregnant women (Shouman et al.), and prescribing cascades related to anticholinergic medications (Trenaman et al.). Authors asked central and potentially clinically-influencing questions related to predicting medication adherence after a myocardial infarction (Campain et al.), repurposing of hydralazine to reduce phelbotomy (Lin et al.), comparison of time to treatment intensifications for diabetes treatments with newer drugs (Roberto et al.), and treatment failure with long-acting antipsychotics (Janzen et al.).

Papers also showcased the ability to assess policy-relevant questions such as the impact of policies on fentanyl prescribing (García-Sempere et al.), opioid use at the end-of-life (Minard et al.), biosimilar uptake of insulin glargine (Hayes et al.), trends in psoriatic arthritis medication use (Faria et al.), and the impact of COVID-19 on psychotropic medication use (Leong et al.). And lastly, this topic area showcased ongoing work related to novel data sources and use of data such as emerging use of Drug utilization research in barzil (Leal et al.), and use of pharmacy and registry data (Serhal et al.).

## Conclusion

With the ubiquity of big data and limitations of RCTs, decision-makers and clinicians are in need of supportive evidence to support the assessment of drug effectiveness, safety, optimal use and policy decisions. This special topic showcased the global power of pharmacoepidemiology for answering this call.

